# Isolated Insular Strokes and Plasma MR-proANP Levels Are Associated with Newly Diagnosed Atrial Fibrillation: A Pilot Study

**DOI:** 10.1371/journal.pone.0092421

**Published:** 2014-03-19

**Authors:** Karl Frontzek, Felix Fluri, Jakob Siemerkus, Beat Müller, Achim Gass, Mirjam Christ-Crain, Mira Katan

**Affiliations:** 1 Institute of Neuropathology, University Hospital Zurich, Zurich, Switzerland; 2 Department of Neurology, University Hospital Basel, Basel, Switzerland; 3 Department of Neurology, University Hospital Zurich, Zurich, Switzerland; 4 University Hospital of Psychiatry, Zurich, Switzerland; 5 Medical University Clinic, Cantonal Hospital Aarau, Aarau, Switzerland; 6 Department of Neurology, University Hospital Mannheim, Mannheim, Germany; 7 Department of Endocrinology, University Hospital Basel, Basel, Switzerland; University Medical Center (UMC) Utrecht, Netherlands

## Abstract

**Introduction:**

In this study, we assessed the relationship of insular strokes and plasma MR-proANP levels with newly diagnosed atrial fibrillation (NDAF).

**Methods:**

This study is based on a prospective acute stroke cohort (http://www.clinicaltrials.gov, NCT00390962). Patient eligibility was dependent on the diagnosis of acute ischemic stroke, absence of previous stroke based on past medical history and MRI, no history of AF and congestive heart failure (cohort A) and, additionally, no stroke lesion size ≥ 20 mL (sub-cohort A*). AF, the primary endpoint, was detected on 24-hour electrocardiography and/or echocardiography. Involvement of the insula was assessed by two experienced readers on MRI blinded to clinical data. MR-proANP levels were obtained through a novel sandwich immunoassay. Logistic-regression-models were fitted to estimate odds ratios for the association of insular strokes and MR-proANP with NDAF. The discriminatory accuracy of insular strokes and MR-proANP was assessed by a model-wise comparison of the area under the receiver-operating-characteristics-curve (AUC) with known predictors of AF.

**Results:**

104 (cohort A) and 83 (cohort A*) patients fulfilled above-mentioned criteria. Patients with isolated insular strokes had a 10.7-fold higher odds of NDAF than patients with a small ischemic stroke at any other location. The AUC of multivariate logistic regression models for the prediction of NDAF improved significantly when adding stroke location and MR-proANP levels. Moreover, MR-proANP levels remained significantly elevated throughout the acute hospitalization period in patients with NDAF compared to those without.

**Conclusions:**

Isolated insular strokes and plasma MR-proANP levels on admission are independent predictors of NDAF and significantly improve the prediction accuracy of identifying patients with NDAF compared to known predictors including age, the NIHSS and lesion size. To accelerate accurate diagnosis and enhance secondary prevention in acute stroke, higher levels of MR-proANP and insular strokes may represent easily accessible indicators of AF if confirmed in an independent validation cohort.

## Introduction

The insular cortex plays an important role in the regulation of the autonomic nervous system. Stimulation of the right insula provokes sympathetic cardiovascular effects, whereas stimulation of the left insula leads to parasympathetic effects.[1] Insular damage has been associated with newly-detected atrial fibrillation (NDAF), atrioventricular blocks and an increased amount of ectopic beats.[2] Furthermore, insular damage is associated with adverse cardiac outcome such as sudden cardiac death and congestive heart failure.[3]–[5].

A-type natriuretic peptides (ANP) have been proposed as biomarkers for atrial fibrillation (AF) and midregional-proANP plasma levels correlate with the duration of AF episodes.[6], [7] There are several sandwich immunoassays commercially available that measure the concentrations of ANP and its precursor protein proANP in human plasma; however, most are prone to fragmentation and may underestimate the release of the precursor due to the early degradation of crucial epitopes at the extreme ends of the molecule. [8], [9] The MR-proANP assay used in this study was designed to detect the mid-region of the prohormone, which is more stable than the N- or C-terminal part of the precursor. [10].

The aim of this study was to analyse the predictive value of isolated infarcts affecting the insular cortex in identifying patients with newly diagnosed atrial fibrillation (NDAF) during the acute hospitalization period from a large, prospectively collected stroke cohort.[11] In a second step, we aimed to investigate the putative role of plasma MR-proANP levels in predicting NDAF and its potential additive value compared to stroke location and other known predictors. Early and accurate identification of stroke patients at high risk for AF is important in selecting candidates for intensive cardiac monitoring such as implantable loop recorders. [12].

## Patients and Methods

### Ethics Statement

This study was based on a prospective cohort study (clinical trial registration at http://www.clinicaltrials.gov, number NCT00390962) at the University Hospital Basel, Basel, Switzerland. The Ethics Committee of Basel, Switzerland, approved the study protocol and informed, written consent was obtained from all patients before enrollment. A complete description of the cohort has been reported previously.[11].

### Description of subjects

**Figure 1:**
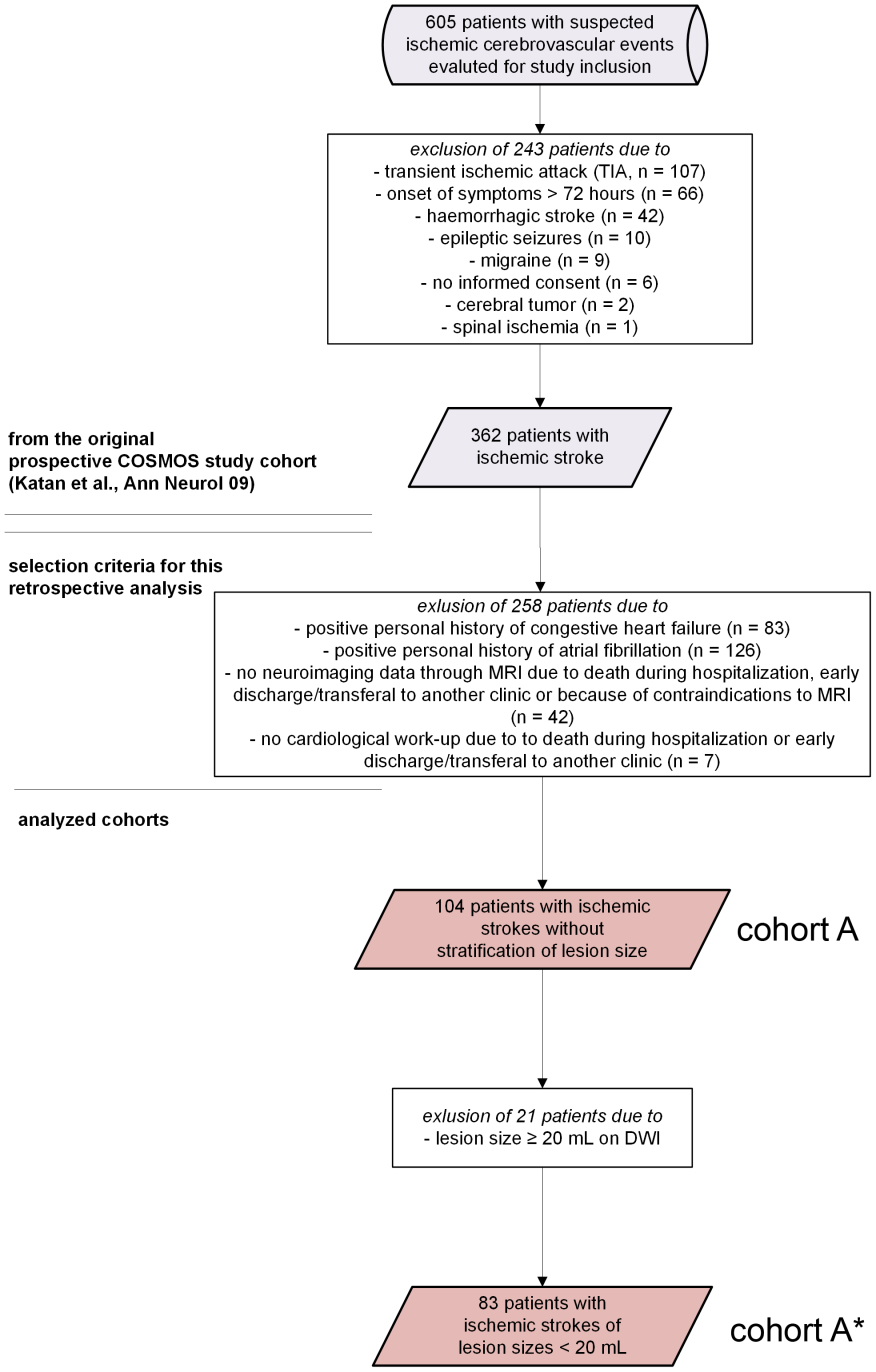
Patient flow chart. The upper part of the figure (as divided by 2 horizontal bars) denotes the study cohort of the original COSMOS cohort as previously described.[11] The lower part of the scheme indicates the eligibility criteria for the cohorts analyzed in this study.

Briefly, from 11/2006 to 11/2007, all patients with a suspected acute ischemic cerebrovascular event were consecutively screened for enrollment in the study. All patients admitted to the emergency department within 72 hours after stroke onset with an acute ischemic stroke defined according to the World Health Organization criteria were included. [13] Stroke severity on admission was assessed by stroke neurologists (MK, FF) using the National Institutes of Health Stroke Scale (NIHSS). All patients underwent a standardized diagnostic workup including brain computer tomography mainly to exclude intracranial hemorrhage, magnetic resonance imaging (MRI), standard 12-lead electrocardiography and/or 24-hour electrocardiography. Echocardiography and neurosonographic studies of the extracranial and intracranial arteries were obtained in order to classify stroke etiology according to the TOAST criteria.[14] Initially, from 605 patients with suspected cerebrovascular events admitted to the emergency department of the University Hospital of Basel, 362 patients with confirmed ischemic stroke were selected (see also *figure 1*). From this cohort, we excluded 258 patients due to positive personal history of congestive heart disease and/or atrial fibrillation (n = 209), those with no imaging data on MRI due to death during hospitalization, early discharge/transferal to another hospital or due to contraindications to MR imaging (n = 42) and those without diagnostic cardiological work-up due to early death during hospitalization or early discharge or transferal to another ward (n = 7). In a second step only patients with ischemic strokes of lesion sizes under 20 mL were selected (see below) yielding a cohort of n = 83.

### Neuroimaging and lesion size stratification

MRI (T1-, T2-, DWI) sequences and MR angiography was performed in all patients using a 1.5-Tesla MR Avanto system (Siemens, Erlangen, Germany). Involvement of the insular cortex was assessed on DWI and T2-weighted images by two experienced readers unaware of clinical data. Stroke lesion volumes on DWI were calculated by the commonly used semiquantitative method.[15] The insula was defined as the cortical region covered by the frontoparietal operculum and the superior temporal plane and overlying the extreme capsule and the claustrum. To obtain an estimate about the volume of the insular cortex, we performed a meta-analysis in the Internet Brain Volume Database for *in vivo* volumetric studies of non-diseased humans.[16] The query yielded 11 studies with a total of 709 investigated subjects. A cumulative meta-analysis using a random effects model for insular volume returned an estimated size of 11.38 mL (95% CI 10.12–12.64 mL). Additionally, previously conducted studies on insular strokes with published lesion sizes showed volumes of insular strokes ranging from around 30 mL [5] to 40-60 mL [4]. Taking these limits as well as our sample size into account we selected a lesion threshold of 20 mL on DWI for insular strokes.

### Assays

Blood was obtained from a venous catheter and plasma was frozen at 70°C. MR-proANP was measured in a blinded batch analysis from all patients using a new sandwich immunoassay (B.R.A.H.M.S. AG), Hennigsdorf/Berlin, Germany), as described in detail elsewhere.[11] In brief, the lower detection limit of the assay is 6.0 pmol/L. The intraassay coefficient of variation was 10%. The interassay coefficient of variation was 10%. In 325 healthy individuals, the range of MR-proANP concentrations was 9.6 to 313 pmol/L. The median was 45 pmol/L (95% confidence interval [CI]: 43.0 to 49.1 pmol/L).

### Statistical analysis

Baseline demographic, clinical observation, laboratory and outcome data were compared between patients with and without insular involvement as well as between those with newly-diagnosed atrial fibrillation and those without. Discrete variables are expressed as frequency (percentage) and continuous variables as medians and interquartile ranges (IQR). For two group comparison, we used Mann–Whitney U test and Chi-squared tests depending on the variable type and distribution of the variable. MR-proANP was ln-transformed to achieve a normal distribution of the variable. One-way ANOVA analysis of variance was used for multi-group comparisons of normally distributed variables with Bonferroni correction of significance for multiple testing. In order to choose the best predictive model to assess the discriminatory value of insular strokes and/or MR-proANP for newly diagnosed atrial fibrillation, we first performed univariate logistic regression analyses for already established predictors of NDAF.[17] Secondly, all variables that were significantly associated (i.e. at a p-value <0.10) with the outcome of interest (i.e. presence of new AF yes/no) in the univariate analysis were then included in a multivariate logistic regression analysis to assess their independent predictive value. The area under the receiver operating characteristic curve (AUC) was calculated to assess the discriminatory value of insular strokes and/or MR-proANP to distinguish patients with NDAF from patients without. The incremental predictive value of insular strokes and/or MR-proANP was tested by comparing the AUC between “model 1” (including all variables that have shown a significant association in the univariate logistic regression model) with the AUC of the nested models 2 and 3 (model 1 *plus* insular strokes =  model 2; or model 3  =  model 2 plus MR-proANP). Analogous calculations for comparison of the AUC in cohort A* are marked as model 1* (isolated insular strokes) and 2* (model 1* plus MR-proANP). For comparisons of these 3 and 2 models, respectively, we used the likelihood-ratio test as recommended [18]. Statistical analyses were undertaken using GraphPad Prism 5 (GraphPad Software, La Jolla, USA),IBM SPSS Statistics 20 (IBM, New York, USA), Comprehensive Meta-Analysis (Biostat, Englewood, USA) and custom-written scripts for MATLAB (MathWorks, Natick, USA). Testing was always two-sided and *P* values less than 0.05 were considered to indicate statistical significance.

## Results

### Study cohorts

We analyzed all patients from a large, prospective stroke study cohort with a known negative history of congestive heart failure and atrial fibrillation that have undergone a diagnostic cardiological work-up on admission and in whom diffusion-weighted imaging was available for volumetric analyses, yielding *cohort A*.[11] In a second step, 21 patients were excluded from cohort A due to a ischemic stroke lesion size ≥ 20 mL on diffusion-weighted imaging to be able to selectively assess the impact of isolated insular strokes, yielding cohort A*. The detailed baseline characteristics of the cohorts A and A* analyzed in this study are given in *table 1*.

**Table 1:** Baseline characteristics of patient cohorts as described in figure 1.

	Cohort A					
	INS	No INS	p-value	NDAF	No NDAF	p-value
number of patients, n	18	86		13	91	
age [y], median (IQR)	72 (57 – 77)	68 (59 – 79)	0.98	71 (67 – 81)	68 (59 – 77)	0.13
NIHSS [pts], median (IQR)	11 (4 – 15)	4 (2 – 7)	0.0019*	5 (2 – 16.5)	4 (2 – 8)	0.28
female sex, % (n)	56 (10)	38 (33)	0.18	38 (5)	42 (38)	0.82
systolic blood pressure [mmHg], median (IQR)	144 (130 – 170)	168 (140 – 183)	0.10	164 (141 – 184)	166 (141 – 185)	0.88
heart rate, median [min^−1^] (IQR)	73 (64 – 89)	68 (75 – 89)	0.66	64 (71 – 78)	76 (67 – 90)	0.31
history of coronary artery disease, % (n)	17 (3)	21 (18)	0.68	15 (2)	21 (19)	0.64
history of arterial hypertension, % (n)	78 (14)	79 (68)	0.78	77 (10)	74 (67)	0.80
lesion size on DWI [mL], median (IQR)	45.3 (9.8 – 100.5)	0.9 (0.1 – 4.1)	< 0.0001*	16.4 (2.8 – 91.1)	1.2 (0.1 – 6.6)	0.0012*
MR-proANP on admission [pM], median (IQR)	151.0 (102.1 – 255.8)	98.5 (68.7 – 169.0)	0.04*	207.0 (122.5 – 341.0)	98.1 (67.5 – 161.3)	0.0004*
Involvement of the insular cortex, % (n)	n/a	n/a		38 (5)	14 (13)	0.03*
Newly-diagnosed atrial fibrillation, % (n)	28 (5)	9 (8)	0.03*	n/a	n/a	

	Cohort A*					
	INS	No INS	p-value	NDAF	No NDAF	p-value
number of patients, n	7	76		8	75	
age [y], median (IQR)	65 (59 – 78)	68 (59 – 79)	0.86	70 (66 – 81)	67 (59 – 78)	0.35
NIHSS [pts], median (IQR)	4 (2 – 8)	4 (1 – 7)	0.70	4 (1 – 10)	4 (2 – 7)	0.89
female sex, % (n)	43 (3)	38 (29)	1	38 (3)	40 (30)	0.89
systolic blood pressure [mmHg], median (IQR)	162 (130 – 184)	163 (143 – 186)	0.57	162 (135 – 184)	168 (143 – 186)	0.66
heart rate, median [min^−1^] (IQR)	87 (65 – 90)	79 (67 – 90)	0.89	74 (65 – 88)	76 (67 – 90)	0.91
history of coronary artery disease, % (n)	14 (1)	21 (16)	0.67	13 (1)	21 (16)	0.56
history of arterial hypertension, % (n)	86 (6)	70 (53)	0.47	75 (6)	71 (53)	1
MR - proANP on admission [pM], median (IQR)	122.0 (87.5 – 174.0)	98.0 (69.1 – 162.0)	0.48	195.5 (113.5 – 398.3)	93.4 (67.5 – 146.5)	0.0031*
Involvement of the insular cortex, % (n)	n/a	n/a		38 (3)	5 (4)	0.0019*
Newly-diagnosed atrial fibrillation, % (n)	43 (3)	7 (5)	0.0019*	n/a	n/a	

(INS  =  insular stroke, NDAF  =  newly diagnosed atrial fibrillation, NIHSS  =  National Institute of Health Stroke Scale, IQR  =  Interquartile Range, CAD  =  coronary artery disease).*p<0.05.

### Isolated insular strokes are independent predictors of newly-diagnosed atrial fibrillation

Of 104 patients taken from the original study cohort that were included for this analysis, 17% (n = 18) suffered from an ischemic stroke involving the insular cortex while it was spared in 83% (n = 86) of patients (for detailed baseline characteristics see also *table 1*, *cohort A*). In univariate logistic regression analysis, stroke severity on admission (as determined by the NIHSS), lesion size on DWI, involvement of the insular cortex and lnMR-proANP levels on admission were significant predictors of newly diagnosed atrial fibrillation in cohort A (p<0.10 for all predictors, *table 2*). Compared to the other predictors, involvement of the insular cortex in ischemic stroke patients failed to improve the predictive value for NDAF (*tables 3*, *4*). However to better investigate the association of insular involvement with NDAF we hence chose to introduce a cut-off for isolated insular strokes based on a cumulative meta-analysis of insular cortex volumes (see also *materials and methods,* [16]) and analyzed the association of insular location with NDAF only in strokes of <20 mL including the insula (i.e. cohort A*). Herein, 8% (n = 7) of the patients suffered from an isolated insular stroke, while the insula was spared in 92% (n = 76) of patients. Of 7 patients with isolated insular strokes, 43% were diagnosed with NDAF (n = 3) while it was diagnosed in 7% of those without isolated insular strokes (n = 4). In univariate logistic regression analysis patients with isolated insular strokes were at 10-fold higher odds for NDAF than those without affection of the insula (OR 10.65, 95% CI 1.85–61.3, p<0.01, *table 5*). In subsequent multivariate logistic regression analysis of cohort A* isolated insular strokes remained an independent predictor of NDAF (OR 13.89, 95% CI 1.79–107.55, p<0.012, *table 6*) with an area under the receiver operating characteristic curve of 0.66, 95% CI 0.43–0.89 (*table 7*). Moreover, adding isolated insular strokes to the predictive model including only MRproANP-levels (in cohort A*), improved the prediction capacity (AUC 0.81 [95% CI 0.68 – 0.93] for lnMR-proANP levels on admission vs. 0.86 [95% CI 0.73 – 0.98] for lnMR-proANP levels on admission *and* isolated insular strokes, p = 0.013, likelihood-ratio test).

**Table 2:** Predictors of newly-diagnosed atrial fibrillation in cohort A.

Risk factor	OR	95% CI	p-Value
**Age [y]**	**1.04**	**0.99** – **1.10**	**0.12**
NIHSS [pts]	1.08	0.99 – 1.18	*0.07^≠^*
Female sex (n)	0.87	0.27 – 2.87	0.82
Systolic blood pressure [mmHg]	1.00	0.98 – 1.03	0.77
Pulse [min^−1^]	0.98	0.95 – 1.02	0.38
Coronary artery disease (n)	0.69	0.14 – 3.38	0.65
Arterial hypertension (n)	1.19	0.30 – 4.71	0.80
**Lesion size [mL]**	**1.01**	**1.00** – **1.02**	0.02^*^
**Involvement of the insular cortex**	**3.75**	**1.06** – **13.20**	0.04^*^
**LnMR-proANP on admission**	**5.03**	**1.86** – **13.56**	0.001^*^

Univariate logistic regression, ***^≠^***p<0.10, *p<0.05.

**Table 3:** Predictors of newly-diagnosed atrial fibrillation in cohort A.

**Model 1**			
**Risk factor**	**OR**	**95% CI**	**p-Value**
NIHSS	1.04	0.93 – 1.15	0.51
Lesion size [mL]	1.01	1.00 – 1.02	0.11
**Model 2**			
**Risk factor**	**OR**	**95% CI**	**p-Value**
NIHSS	1.03	0.92 – 1.15	0.60
Lesion size [mL]	1.01	1.00 – 1.02	0.19
Involvement of the insular cortex	2.28	0.57 – 9.20	0.25
**Model 3**			
**Risk factor**	**OR**	**95% CI**	**p-Value**
NIHSS	0.97	0.85 – 1.11	0.69
Lesion size [mL]	1.01	1.00 – 1.02	0.14
Involvement of the insular cortex	1.04	0.93 – 1.15	0.51
LnMR-proANP on admission	4.69	1.58 – 13.88	0.005*

Multivariate logistic regression, *p<0.05.

**Table 4:** Comparison of the area under the receiver operating characteristic curve of each model in cohort A.

Model	AUC	95% CI	p-value for comparison
1	0.69	0.54 – 0.83	n/a
2	0.74	0.6 – 0.88	0.26
3	0.85	0.75 – 0.94	*0.002* *

*p<0.05**.**

**Table 5:** Predictors of newly-diagnosed atrial fibrillation in cohort A*.

Risk factor	OR	95% CI	p-Value
Age [y]	1.03	0.97 – 1.09	0.34
NIHSS [pts]	1.02	0.89 – 1.17	0.74
Female sex (n)	0.90	0.20 – 4.05	0.89
Systolic blood pressure [mmHg]	1.00	0.97 – 1.03	0.79
Pulse [min^−1^]	1.00	0.95 – 1.04	0.91
Coronary artery disease (n)	0.53	0.06 – 4.60	0.56
Arterial hypertension (n)	1.25	0.23 – 6.65	0.80
**Isolated insular stroke**	**10.65**	**1.85** – **61.30**	***0.008^#^***
**LnMR-proANP on admission**	**5.18**	**1.60** – **16.75**	***0.006^#^***

Univariate logistic regression, ^#^p<0.05.

**Table 6:** Predictors of newly-diagnosed atrial fibrillation in cohort A*.

Model 1*			
**Risk factor**	**OR**	**95% CI**	**p-Value**
Isolated insular stroke	10.65	1.85 – 61.30	*0.008* ^#^
Model 2*			
**Risk factor**	**OR**	**95% CI**	**p-Value**
Isolated insular stroke	13.89	1.79 – 107.55	*0.012* ^#^
LnMR-proANP on admission	6.00	1.61 – 22.37	*0.008* ^#^

Multivariate logistic regression, ^#^p<0.05.

**Table 7:** Comparison of the area under the receiver operating characteristic curve of each mode in cohort A*.

Model	AUC	95% CI	p-value for comparison
1*	0.66	0.43– 0.89	n/a
2*	0.86	0.73 – 0.98	0.013#

# p<0.05.

### Plasma MR-proANP measured on admission is an independent predictor of newly-diagnosed atrial fibrillation

**Figure 2:**
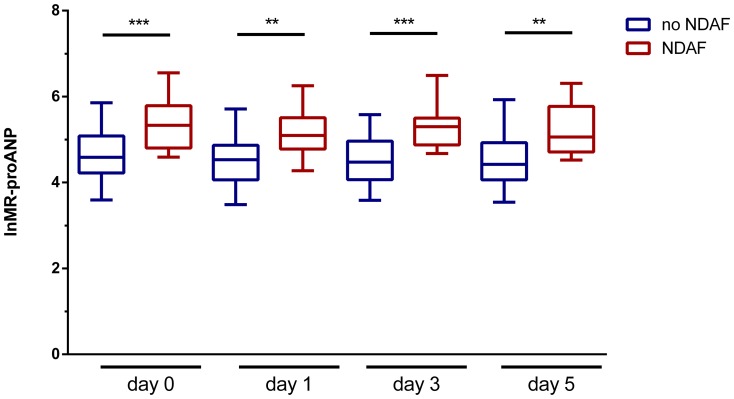
Timecourse of plasma MR-proANP levels. Plasma lnMR-proANP levels are plotted on the ordinate, lnMR-proANP levels were significantly different between patient groups throughout the monitored period (day 0 denotes the day of admission; ** p<0.01, *** p<0.001, one-way ANOVA with Bonferroni’s multiple comparisons post-hoc test). The abscissa denotes the time points of plasma MR-proANP levels during the hospitalization period in patients with no newly-diagnosed atrial fibrillation (no NDAF, blue boxes) and those with newly-diagnosed atrial fibrillation (NDAF, red boxes). Horizontal bars indicate group means with error bars denoting 95% CI.

In univariate logistic regression analysis, lnMR-proANP levels on admission showed a significant association with NDAF (cohort A, OR 5.03, 95% CI 1.86–13.56, p = 0.001, *table 2*). In subsequent multivariate logistic regression models, lnMR-proANP levels on admission were independently associated with NDAF (OR 4.69, 95% CI 1.58–13.88, p = 0.005, *table 3* *)*. LnMR-proANP levels on admission improved the predictive accuracy for NDAF in cohort A as determined by the comparison of the area under the receiver operating characteristic curve (p = 0.002, likelihood-ratio test, *table 4*), an effect that was also observable in cohort A* (p = 0.013, likelihood-ratio test, *table 7*). A cut-off value of 98.35 pM for plasma levels of MR-proANP corresponded to a detection sensitivity for NDAF of over 92.3%, thus in patients with levels below 98.4 pM atrial fibrillation is highly unlikely. A specificity of >98.9% was obtained at plasma levels over 538.5 pM, thus in these patients detection of atrial fibrillation is almost certain. LnMR-proANP levels stayed significantly different between patients with NDAF and those without throughout the monitored time course (p<0.001, day 0 [i.e. the day of admission]; p<0.01, day 1; p<0.001, day 3 and p<0.01, day 5; one-way ANOVA with Bonferroni’s post-hoc adjustment of significance, *figure 2*).

### The association of MR-proANP and insular strokes

Although patients with strokes involving the insular cortex from cohort A did have higher MR-proANP levels on admission than those without (insular strokes 151.0 pM IQR 102.1–255.8 vs. non-insular strokes 98.5 pM IQR 68.7–169.0, p = 0.04, Mann Whitney U Test, *table 1*), after adjusting for lesion size, MR-proANP levels were no longer associated with isolated insular strokes (in cohort A*).

## Discussion

In this study, we found that patients with isolated insular strokes (i.e. ischemic strokes of the insula with a volume <20 mL on DWI) were at 10.7-fold higher odds for NDAF than those without. Insular location significantly improved the predictive model to identify NDAF when compared with other prognostic factors. Moreover we found that higher levels of lnMR-proANP were associated with 5.2-fold higher odds of newly detected AF. In addition, the inclusion of lnMR-proANP levels in to the existing predictive model (including insular stroke) further improved differentiation between patients with NDAF and patients without NDAF.

In line with our findings, involvement of the insula in ischemic stroke patients was previously shown to be associated with severe cardiac derangement leading to complex ECG abnormalities such as premature ventricular contractions, non-sustained ventricular tachycardia and others when compared to ischemic stroke patients without insular involvement. Herein, especially right-sided insular lesions were associated with newly detected cardiac arrhythmia.[5] In another study with a smaller patient cohort, a greater increase in plasma norepinephrine concentrations was observed in patients with insular infarctions when compared to non-insular stroke patients, thus suggesting a possible direct connection of the insular lesion to activation of the sympathetic nervous system.[19] Lesion sizes of investigated subjects in the former study added up to 30 mL on average, while the latter did not adjust for lesion size at all. However, a cumulative analysis of insular volume in non-diseased patients yielded an average size of 11.38 mL (95% CI 10.12 – 12.64 mL, [16]). Above-mentioned lesions (i.e. >30 mL) are thus highly likely to affect other brain structures in the immediate anatomical neighborhood that are also described as regulators of the brain-heart-axis amongst others the caudate nucleus and the ventromedial prefrontal cortex.[20] In contrast to our study where lesion sizes was restricted to 20 mL (i.e. cohort A*) the results of these other studies might lack specificity.

Patients with AF, especially when undiagnosed and/or untreated are more prone to cardiovascular complications such as myocardial infarctions and thromboembolic events such as strokes.[21] In a prospective study with a larger cohort, patients with ischemic infarcts of the left insula were followed-up over 1 year and screened for sudden cardiac death, myocardial infarction and congestive heart failure. When compared to patients with non-insular infarcts and TIAs, left insular strokes were associated with an increased risk for adverse cardiac outcome as well as impaired cardiac wall motion. The exclusion of TIAs, however, did not yield a significant difference.[3] Increased QTc interval and the presence of left bundle branch block were also associated with higher vascular mortality rates in patients with right-sided insular infarcts.[4] These clinical implications of disturbances of the brain-heart-axis should encourage for heightened sensitivity in the acute hospitalization period in regards to selection of patients especially vulnerable towards cardiac abnormalities that may profit from a more stringent cardiological work-up and/or early detection devices amongst others implantable loop recorders. [12].

Since it is not possible to ascertain whether our patients with newly diagnosed AF had already experienced AF episodes before the stroke, we cannot make any inferences regarding causality. However, it is biologically plausible that an insular stroke in patients with paroxysmal subclinical AF or with other pre-existing but clinically unapparent cardiac dysfunction may, via activation of the sympathetic nervous system, trigger longer episodes of AF or may convert into AF, which then can be detected by monitoring. Thus, insular lesions in acute stroke patients with elevated plasma MR-proANP levels should prompt an aggressive cardiac work-up and potentially even outpatient monitoring to increase the yield for the detection of AF also in patients where the stroke etiology was not believed to be cardioembolic.

In a pilot study, mid-regional ANP precursor protein has been proven to represent a promising biomarker for determining the duration of AF episodes. More specifically, MR-proANP levels were higher in patients with AF episodes lasting longer than 48 hours compared to patients with episodes of less than 48 hours duration (321.7 IQR 236.4–425.6 versus 144.0 IQR 129.2–213.7 [pmol/L].[7] In our study, patients with elevated lnMR-proANP levels had 5.03-fold greater odds of having newly-diagnosed atrial fibrillation. Levels of brain natriuretic peptide (BNP) have been shown to be higher in patients where NDAF was documented during the hospitalization period. This association was independent of age, NIHSS and female gender.[22] In another more recently published retrospective analysis from the *Warfarin-Aspirin recurrent stroke study*, patients with plasma concentrations of amino-terminal pro-BNP (NT-proBNP) over 750 pg/mL seemed to be at lower risk for subsequent events (i.e. recurrent ischemic stroke or death over 2 years) when treated with anticoagulants other than antiplatelet agents.[23] These findings underscore the role of natriuretic peptides as serum markers of impending cardiac arrhythmias and assist in their clinical management. Although in our study, a significant difference in MR-proANP levels was detected between patients from cohort A with ischemic strokes involving the insula and those without, insular strokes failed to be predictive for MR-proANP levels in linear regression analyses with and without lesion size stratification (*data not shown*). This suggests that the association of insular strokes with new onset of AF is not in the same causal pathway as MR-proANP and AF. Another explanation is that we were underpowered and therefore unable to find any type of mediation.

Some limitations merit attention. First, newly-diagnosed atrial fibrillation in insular stroke patients was not the primary endpoint of the original study cohort; however, all data were prospectively collected and biomarker measurements were pre-planned and assessed in a blinded manner. Second, we did not collect information on NDAF after discharge, thus we may have missed some patients with insular strokes and/or elevated MR-proANP levels that did not show signs of new-onset atrial fibrillation during their hospitalization but suffer from intermittent atrial fibrillation. [24] Hence this potential misclassification is more likely to underestimate the association of insular strokes with NDAF (i.e. bias towards the null-hypothesis) because in the group without NDAF some patients might have had atrial fibrillation thus raising the number of false negatives. Thirdly, due to sample size limitations, we did not assess insular substructures, such as anterior/posterior division or laterality, although some studies have investigated this complex and controversial topic of specifically assigned parts of the insula.[25] Finally, the results of this pilot study should be interpreted with caution due to the relatively small numbers thus an external validation in an independent larger cohort is necessary. Still, these promising results should propagate further investigation with larger patient cohorts since our findings could be of practical clinical value for the early identification of AF patients which might benefit from oral anticoagulation.
